# Semi-automatic recognition of marine debris on beaches

**DOI:** 10.1038/srep25759

**Published:** 2016-05-09

**Authors:** Zhenpeng Ge, Huahong Shi, Xuefei Mei, Zhijun Dai, Daoji Li

**Affiliations:** 1 State Key Lab of Estuarine & Coastal Research, East China Normal University, Shanghai, China

## Abstract

An increasing amount of anthropogenic marine debris is pervading the earth’s environmental systems, resulting in an enormous threat to living organisms. Additionally, the large amount of marine debris around the world has been investigated mostly through tedious manual methods. Therefore, we propose the use of a new technique, light detection and ranging (LIDAR), for the semi-automatic recognition of marine debris on a beach because of its substantially more efficient role in comparison with other more laborious methods. Our results revealed that LIDAR should be used for the classification of marine debris into plastic, paper, cloth and metal. Additionally, we reconstructed a 3-dimensional model of different types of debris on a beach with a high validity of debris revivification using LIDAR-based individual separation. These findings demonstrate that the availability of this new technique enables detailed observations to be made of debris on a large beach that was previously not possible. It is strongly suggested that LIDAR could be implemented as an appropriate monitoring tool for marine debris by global researchers and governments.

Global anthropogenic debris pervades the earth’s marine ecosystems and has exponentially increased in recent decades^1^,^2^,^3^. Most marine debris from various activities, such as tourism, agriculture, fisheries and industry, is likely to move away from its original source and ultimately deposits on a marine shore or floats in the sea due to river transport, waves and tidal actions^4^,^5^. Vast amounts of marine debris cause severe challenges to the global environment by posing immense threats, especially on marine organisms, ecological processes and marine economies^6^,^7^. Many countries and international organizations (NOAA, OSPAR) began to monitor marine debris in the 1970s and have since developed standardized programs for monitoring and assessing marine debris^8^,^9^,^10^. However, most of these routine investigations are primarily based on in-site surveys. These manual observation tools are costly, time-consuming, lack specific information and may be influenced by the subjectivity of the operators^11^. To better understand the sources, types, potential pathways and temporal trends of marine debris, environmental monitoring tools that are capable of directly and appropriately obtaining valuable and principle records of changes in marine debris are urgently needed^12^,^13^,^14^.

Recently, researchers developed an imaging method to detect colored, macro-plastic debris on beaches based on a webcam located in Japan that makes continuous automatic monitoring possible^15^,^16^. However, many other types of marine debris apart from macro-plastic debris are a matter of concern in the context of increasing global pollution. To easily and rapidly capture the three-dimensional (3D) spatial information and actual physical size of different marine debris deposited on the shore, new techniques are urgently needed.

LIDAR (light detection and ranging), an active surveying technology, provides point-cloud datasets^17^. Point-cloud means a cluster of points look like cloud, which is a vector dataset different from raster image^18^. These point-cloud datasets, as the digitized and discretizing expression of the real objects in computer by means of laser scanner, reflect the spatial 3D features of the actual objects. Compared to traditional surveying techniques, LIDAR technology has higher spatial resolution and superior expression ability in 3D space based on point-cloud datasets^18^. Until recently, LIDAR technology had been primarily applied in fields such as topographic analysis, medical science, forest science, robot technology and archaeology, among others^19^,^20^,^21^,^22^,^23^,^24^,^25^. Full-waveform LIDAR data have been studied and utilized since 1990s^26^. The data are recorded by the backscattering signal of echoes, which originate from the interaction between a laser pulse and an object^26^. Once the full-waveform data are decomposed, different echo amplitudes, echo widths and echo sequences for each scanned object with its available spatial positional information are obtained, which can be further used for classification^18^. Therefore, LIDAR can be used to identify different marine debris with different full-waveform features. However, the LIDAR technique has yet to be used to detect marine debris, especially not the application of full-waveform LIDAR data for monitoring marine debris.

Accordingly, this study proposes a semi-automatic method to analyze the amount and morphology of marine debris on the basis of the LIDAR technique. The idea of semi-automatic method mainly depends on laser scanner and computer with less human interventions, which agrees well with “supervised classification”^27^. As expected, LIDAR technology, which is a cost-effective and highly precise tool for quantifying the number and configuration of marine debris, could contribute to improving traditional monitoring methods.

## Results

### Data collection by LIDAR

**Figure 1:**
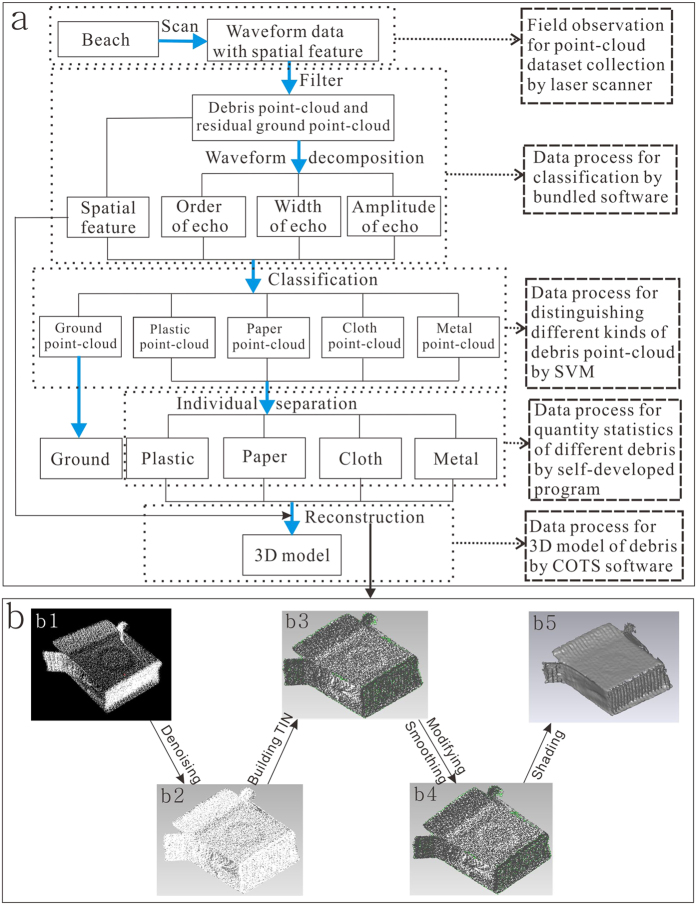
Study workflow. (**a**) The steps of the whole research. (**b**) The steps of 3D modeling reconstruction. The figure was created in CorelDRAW Graphics Suite X5.

In our experiment, a point-cloud dataset containing approximately 9.6 × 10^7^ points were captured in a 5.4 × 10^4^ m^2^ area over 13 minutes using a laser scanner on Nanhui beach (Fig. 1a; Supplementary, Figs S1 and S2b). Then, the area containing debris was extracted from the scanned area, which included 2.5 × 10^7^ points (Supplementary, Fig. S2c). After filtering, the number of points was reduced to 4.3 × 10^6^ (Supplementary, Fig. S2d).

### Identification of debris

**Figure 2 f2:**
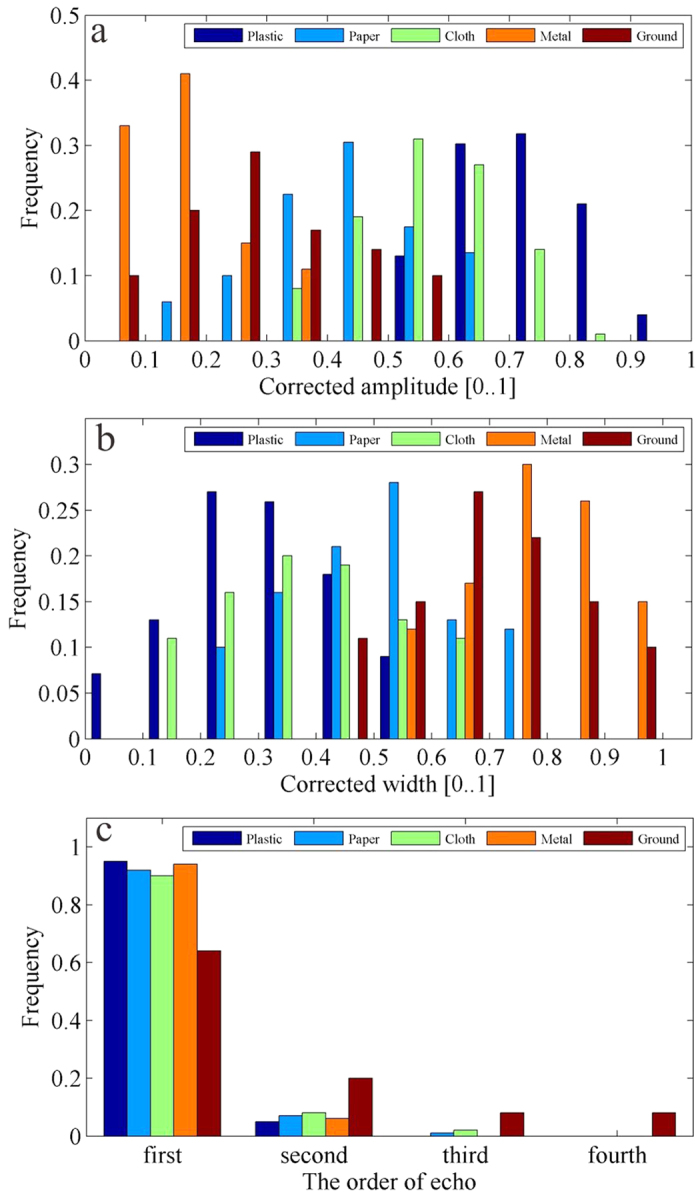
Frequency histograms of the waveform features of points in the simulated experiment at Nanhui beach. (**a**) Frequency histogram of the corrected amplitude [0, 1]; the amplitudes are normalized by the maximum value. (**b**) Frequency histogram of the corrected width [0, 1]; the widths are normalized by the maximum value. (**c**) Frequency histogram of the echo order. The figure was created in Matlab R2012a.

The frequency histogram of the full-waveform marine debris features from Nanhui beach was obtained after the full-waveform data were filtered, decomposed, and classified based on the aforementioned methods (Figs 1 and 2a–c). The echo amplitude of the different marine debris differed in the distribution regions, as characterized by distinct dominants (Fig. 2a). The echo amplitude was primarily distributed in the 0.5–1 range, with a dominant peak frequency between 0.6 and 0.8 for the plastic debris, and in the 0.3–0.8 range, with a peak frequency between 0.5 and 0.7 for the cloth debris (Fig. 2a). In addition, the echo width for the different debris, such as paper and cloth, had their own characteristics (Fig. 2b). However, no significant difference in the echo order was observed in most of the marine debris (Fig. 2c).

**Figure 3 f3:**
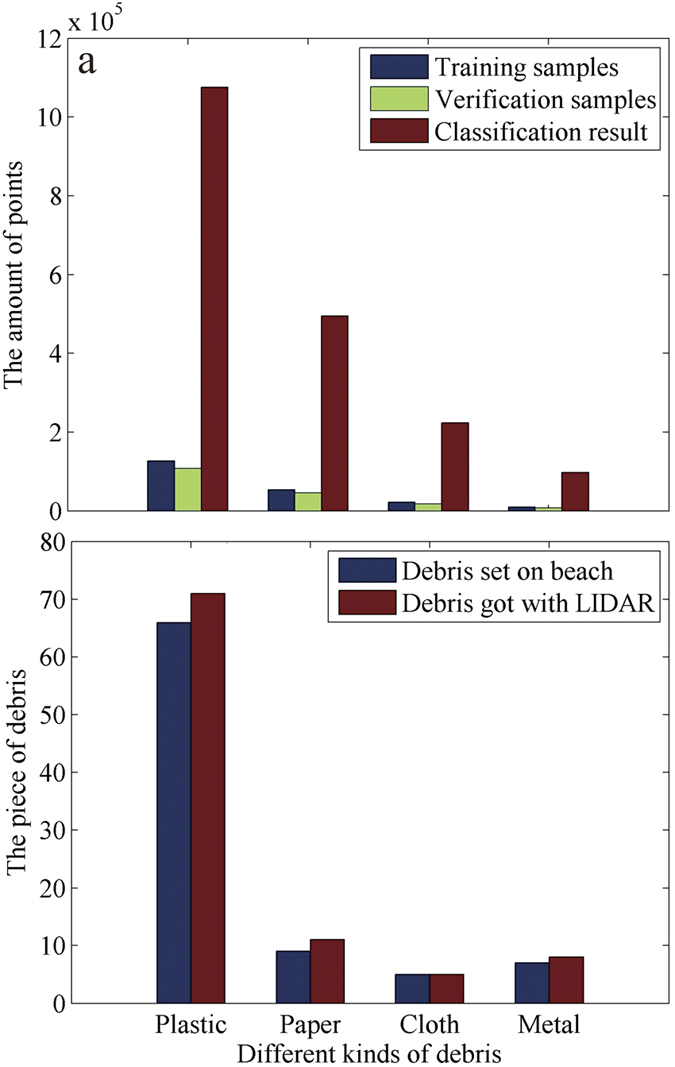
The development of identification methods for marine debris using LIDAR. (**a**) Classification of points based on SVM; the information of classification accuracy can be found in Table S1. (**b**) Matches of the number of predetermined debris with the debris identified by LIDAR. The figure was created in Matlab R2012a.

The echo amplitude, echo width, and echo order features of the marine debris were further classified using SVM. As shown in Fig. 3a, the marine debris points on Nanhui beach were sorted into plastic, paper, cloth, and metal with their corresponding point statistics. The sorted marine debris points were further identified as different types of marine debris via individual separation (Fig. 3b). The LIDAR technology was found to be comparable to manual identification for detecting the quantity of marine debris (Fig. 3b).

### Reconstruction of the 3D model of marine debris

**Figure 4:**
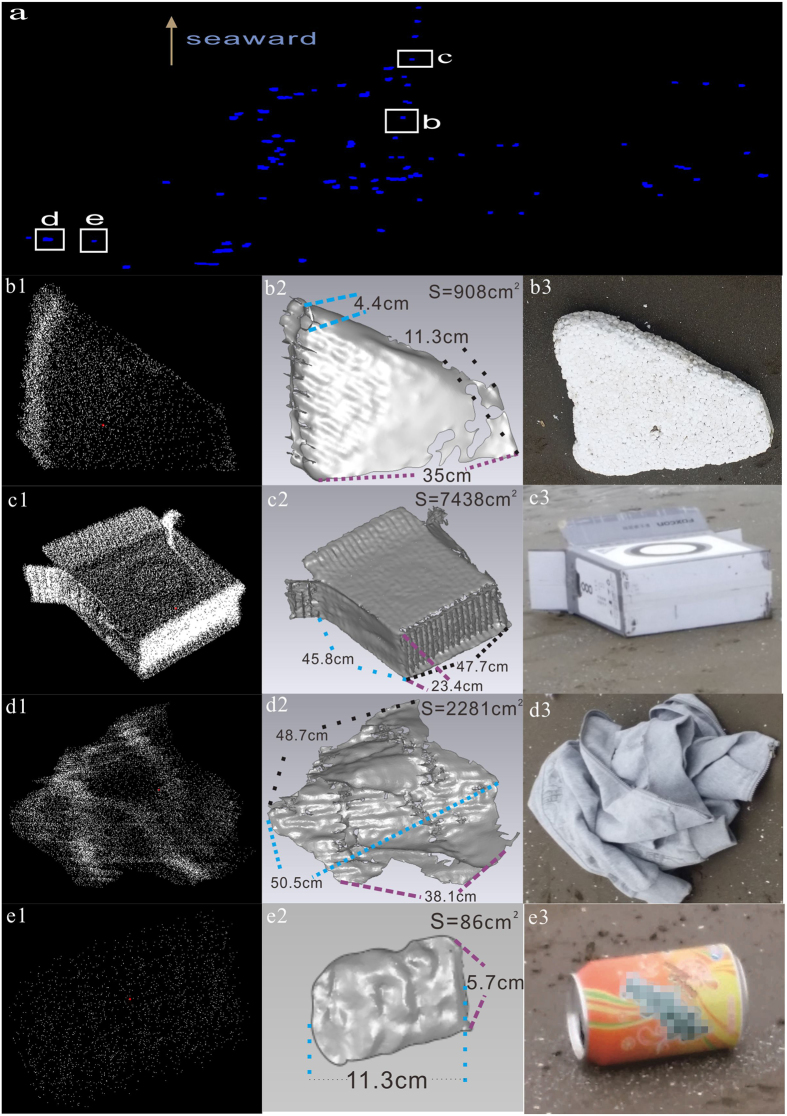
Distribution and reconstruction of debris in the simulated experiment at Nanhui beach. (**a**) Distribution of identified debris on the beach. The boxes (**b**–**e**) represent plastic (b1–b3), paper (c1–c3), cloth (d1–d3) and metal (e1–e3). (**b**–e1), point-clouds of individual debris; (**b**–e2), 3D models of individual debris; S is the surface area of the debris, and the distance between two points is the curve length on the surface; (**b**–e3), photos of real objects. The figure was created in Geomagic Studio 2013 and CorelDRAW Graphics Suite X5.

Although the distribution of marine debris can be distinguished from afar, its 3D geometrical characteristic is indistinguishable (Fig. 4a). Thereof individual point-cloud, such as plastic, metals will go through a series of process, including denoising^28^, triangulate irregular network (TIN) building^29^, modifying and smoothing^30^,^31^, and shading^32^ (Fig. 1b). Thereafter, 3D models of those objects were reconstructed to describe the configuration features of different marine debris (Fig. 4b–e2). Compared with the real objects (Fig. 4b3–e3), the 3D models displayed the basic geometry of marine debris well. Moreover, the 3D model of larger object (Fig. 4c2) is more lifelike than that of smaller object (Fig. 4e2) while that of regular object (Fig. 4c2) is more realistic than that of irregular object (Fig. 4d2). Accordingly, the large and regular object perform better than those small and irregular one in 3D reconstruction of our research.

### Validation of LIDAR in debris investigation

**Figure 5:**
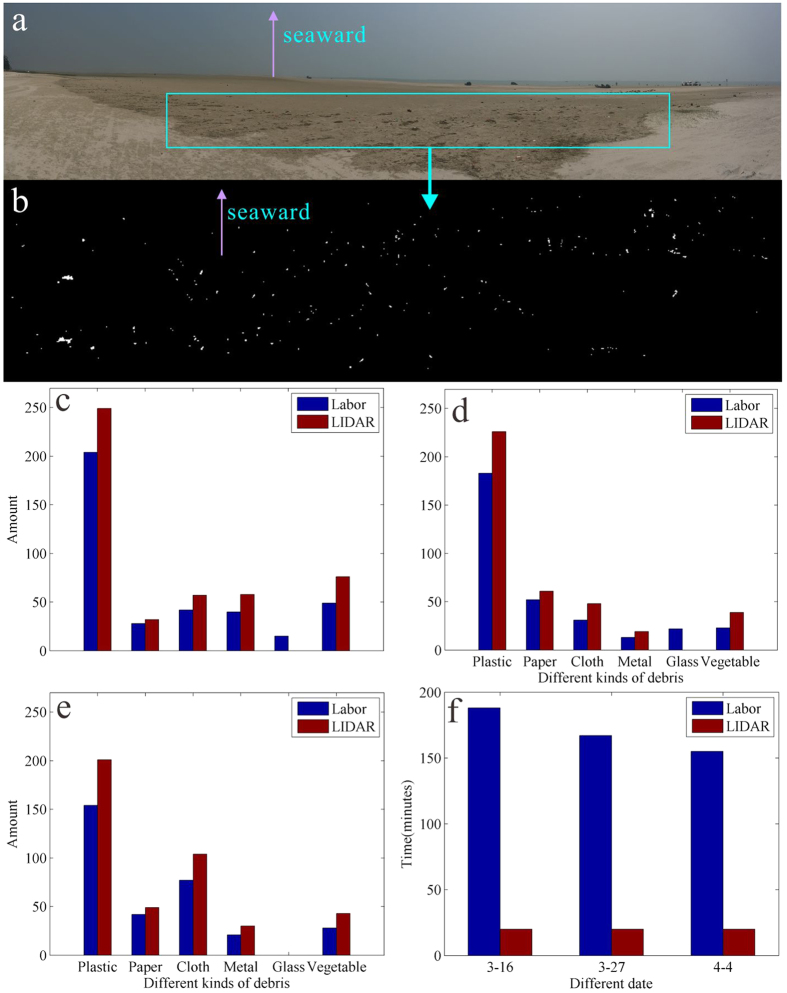
Validation of the semi-automatic recognition method for marine debris on Beihai beach. (**a**) Photo of the beach on March 16, 2015. The box represents the study area where the statistical data of the debris were manually obtained. (**b**) Distribution of debris identified by LIDAR in the study area. (**c**–**e**) Comparisons of the types and quantities of debris for the manual and LIDAR methods. (**f**) Comparison of the time consumed for the manual and LIDAR methods. The figure was created in RiSCAN PRO 1.7.8 and CorelDRAW Graphics Suite X5.

In the designed experiment on the Nanhui beach, 87 objects were set, among which 72 were detected and reconstructed, indicating a high accuracy rate of 82.8%. Based on the Nanhui beach workflow (Fig. 1a), three more field studies were conducted to detect marine debris classification at a beach along Beihai, China (Fig. 5a,b). LIDAR identified much more debris for each type than the manual method, with the exception of glass debris (Fig. 5c–e). Furthermore, the scanner obtained the data within 20 minutes, which is much shorter than the manual method. Although the identification of glass debris doesn’t work, the mean accuracy of LIDAR is 75.4%. According to the results of Nanhui beach and Beihai beach experiments, marine debris detection with LIDAR has low time consuming and high recognition efficiency when compared to that of manual method.

## Discussion

As each type of landscape has its own spectral information, each type of marine debris has different backscattering characteristics in response to a laser, such as echo amplitude, width, and sequence^33^. Accordingly, we can benefit from the use of LIDAR technology to scan marine debris and further extract and classify marine debris according to its waveform features, which have been verified by our controlled test and three cruise field validation tests (Figs 3 and 5). While the world’s beaches primarily consist of sand without obstructions, such as vegetation, the types of marine debris worldwide are similar. Therefore, LIDAR technology can be used extensively to obtain marine debris information distributed on beaches worldwide. Furthermore, LIDAR can be used to reconstruct the configuration of marine debris based on point-clouds. A 3D model of marine debris is similar to the real object, which presents the possibility of reconstructing the state of beach debris using virtual reality technology (Fig. 4).

However, glass debris was not detected successfully in validation experiments. Glass and sand each contain SiO_2_ as their major chemical component, which may be the key reason for the misclassification of glass. Furthermore, being able to discriminate between different types of marine debris also depends on the point density of point-cloud, which is determined by the distance between the machine and the object. For example, in our experiment at Beihai beach, for those items that were located 115 m from the scanner, the smallest identified object was a plastic bottle with a length of 8.5 cm. In addition, when the point density of point-cloud is fixed more points can be scanned in large objects, which will generate more realistic 3D model^32^ (Fig. 4c1–c3, [Fig f2], [Fig f3],e1–e3, [Fig f2], [Fig f3]). Besides that, compared with regular object, the irregular one has more complex surfaces to be simulated and more points for surface fitting in 3D reconstruction^34^, accordingly the 3D model of regular object is more vivid in our research (Fig. 4c1–c3, [Fig f2], [Fig f3],d1–d3, [Fig f2], [Fig f3]).

Compared to manual counts, LIDAR has observable advantages in time and effort saving. In Fig. 5f, it took approximately 3 hours to complete the marine debris identification, and personal error is inevitable during the work. LIDAR technology provides excellent results within 20 minutes. Additionally, in this study, the scanner is fixed on a tripod, and the effective coverage area depends on the instrument. If the laser scanner were to be installed on a mobile vehicle combined with an inertial navigation system, the coverage of scanning would be greatly widely to obtain massive amounts of data along with location information. Thus, LIDAR technology could be used extensively in monitoring marine debris on a large scale.

Despite some the successful achievements of some researchers^11^,^35^,^36^,^37^, photography is limited by light, which leads to invalidation in foggy weather and at night. Furthermore, a single webcam can only express 2D features of marine plastic debris, which fails to identify the thickness of the plastic, let alone classify different types of marine debris. LIDAR is based on an active laser pulse that is capable of obtaining 3D geometric features around the clock. Obviously, LIDAR technology is significantly superior for identifying material types, spatial information and geometric features of MAD. Predictably, the informatization of marine debris monitoring by LIDAR will become more mainstream in the future.

Many large-scale programs have been developed around the world to detect marine debris on beaches. In these investigations, intensive manual work has been conducted^38^. If LIDAR technology was used in regular surveys of marine debris, the efficiency of information acquisition could be substantially improved. Moreover, it is often difficult to obtain data with a high time resolution due to time constraints. With the spatial and geometrical information from LIDAR, the movement and deformation of marine debris can easily be investigated and understood.

## Conclusions

In this study, we present a new technique for the semi-automatic recognition of anthropogenic macro-debris on a beach using light detection and ranging (LIDAR) technology. We first reconstruct the three-dimensional models of different debris types using the individual point-cloud data obtained through individual separation, which can maximize the revivification of debris on the beach. Additionally, this technique, which was tested in a controlled test with three cruise field validations, is extremely less laborious and less time consuming compared with manual methods. We believe that this new technique will enable detailed debris observations to be made on spatially extensive beaches, a feat that was previously not possible. The result is that new opportunities are provided to study the spatial patterns, temporal changes and accumulation of debris, vastly increasing the accuracy of monitoring environmental debris. This technique can serve as an appropriate debris monitoring tool among global researchers and governments.

## Methods

### Experimental preparation

The experiment was conducted at Nanhui beach (Shanghai) on January 1, 2015, in fair weather, Nanhui beach, located at the junction of the South Channel and the adjoining land, is an edge-typed spit. The beach primarily consists of fine sand without any debris due to northern wind effects, which result in an ideal area for this experiment. Marine debris, including plastic, carton, clothing, and metal, was artificially counted in advance and then placed on the beach 100 meters in front of a laser scanner.

### Beach Scanning

A RIEGL VZ-4000 terrestrial laser scanner was rotated 0.004 degrees horizontally and 0.003 degrees vertically (http://www.riegl.com/nc/products/terrestrial-scanning/produktdetail/product/scanner/30/). The scanner was stationed on the seawall with a stable tripod (Supplementary, Fig. S2a). After the laser scanner operated for approximately 13 minutes, the full-waveform data, including the beach surface and marine debris samples with their spatial position information, were taken. The full-waveform data consisted of discrete points, the spatial resolution of which ranged from 0.2 cm near the scanner to 6.2 cm along the seaside that were automatically uploaded onto a computer. Further extraction information of the marine debris from discrete points was performed using a RiWAVELib platform (http://www.riegl.com).

### Data filtering

The marine debris on the beach and surrounding beach ground surface has different point-cloud elevations. Most of the beach ground surface points that were lower than the marine debris could be filtered while data from the debris and residual ground points were obtained (Supplementary, Fig. S2d).

### Full-waveform data decomposition

The full-waveform LIDAR data of the debris and residual ground points were further decomposed based on Gaussian decomposition^26^. The filtered full-waveform data were decomposed into echo order, echo width and echo amplitude (Supplementary). Then, a radiometric correction was used for the removal of the distance effect on the echo amplitude^39^,^40^,^41^ (Supplementary). The full-waveform LIDAR data of the marine debris were represented by the above three full-waveform features and spatial position information.

### Data classification

To identify different types of marine debris on the beach, we applied the support vector machine (SVM) to decompose the full-waveform data^42^. The SVM is a common supervised mathematic classification method that has been widely used in geoscience and environmental research^43^. Using the SVM, discrete points with similar properties are classified into a single group (Supplementary).

### Individual separation

After the SVM process, the decomposed full-waveform LIDAR data of the debris and residual ground points could be grouped into several species, including ground surface point-cloud, plastic point-cloud, paper point-cloud, cloth point-cloud, and metal point-cloud. The point-clouds obtained, except for the ground surface point-cloud, were further separated into individuals using spatial position information and a scanline seed fill algorithm^44^. Additionally, objects with points that were less than the threshold value were ignored to reduce misclassification on the edge of the objects (Supplementary).

### Reconstruction

Ultimately, the classified debris points obtained via individual separation were reconstructed based on GeomagicStudio2012 (www.geomagic.com). (1) Denoising, due to the influence of instruments and the environment, the obtained point-cloud usually carries certain noise points, which may result in rough surface of 3D model^28^. For reducing such phenomenon, denoising is performed based on Laplace operator coupling with scale-space theory^45^ (Fig. 1b(b1,b2)); (2) TIN building, with the help of Delaunay triangulation^29^, the point-cloud dataset is transformed to TIN for approximating the surface of the real object, and the density of the TIN is adjusted to achieve the optimal effect^46^ (Fig. 1b(b2,b3)); (3) Modifying and smoothing, based on the triangulation model, the non-manifold triangulations are modified (deleted or merged) to improve the TIN structure^30^ while smoothing is conducted to minimize crease angles between polygons and remove spikes^31^ (Fig. 1b(b3,b4)); (4) Shading, till now, the 3D model is still in wire frame from the visual point. The flat shading method is therefore used to enhance the third dimension of the 3D model^32^ (Fig. 1b(b4,b5)). Thereafter, the geometric properties of marine debris can be obtained.

### Validation experiment

To test the validity and generalizability of the method in this paper, we performed an experiment three times on the touristic beach of Beihai, Guangxi province, in fair weather. The beach is open to the South China Sea, with a vast amount of debris sourced from the ocean and tourists. Each experiment was conducted in a 150 × 50 m^2^ area with different marine debris types. A laser scanner was operated for approximately 20 minutes to obtain the full-waveform data. Meanwhile, a manual assessment was performed in the same area, recording the marine debris quantity and type for comparison purposes (Supplementary, Figs S3–S5).

## Additional Information

**How to cite this article**: Ge, Z. *et al.* Semi-automatic recognition of marine debris on beaches. *Sci. Rep.* **6**, 25759; doi: 10.1038/srep25759 (2016).

## Supplementary Material

Supplementary Information
