# Supplementary material for: Semi-automatic recognition of marine debris on beaches
Source: Sci Rep. 2016 May 9;6:25759. doi: 10.1038/srep25759 (PMC4860581; doi:10.1038/srep25759)
Supplement: Supplementary Information [file srep25759-s1.pdf]

## Supplementary Information for:

# Semi-automatic recognition of marine debris on beaches

Zhenpeng Ge[1], Huahong Shi[1], Xuefei Mei[1], Zhijun Dai[1], Daoji Li[1]

[1]State Key Lab of Estuarine & Coastal Research, East China Normal University, Shanghai, China

**Corresponding author**

Zhijun Dai

State Key Lab of Estuarine & Coastal Research,

East China Normal University,

Shanghai,

China

Email: zjdai@sklec.ecnu.edu.cn;

Tel: +86 21 62233458;

Fax: +86 21 62546441

These authors contributed equally to this work.

## Supplementary data

### The results of SVM in Nanhui beach

In this study, a SVM is used for the classification of points and the training sample is selected manually. In theory, we can achieve better results if the selected points are more uniform in space and species. Accordingly, we attempt to obtain acceptable results (shown in Table S1). The total accuracy = 80.34%, and Kappa coefficient = 0.7542.

### The introduction of LIDAR system

The system of LIDAR contains the durable laser scanner and bundled software. There are many types of LIDAR system, such as airborne LIDAR system, Satellite-bone LIDAR system, ship borne LIDAR system and terrestrial LIADR system (TLS). In this research, we applied the TLS which is featured by low-cost compared to the types of LIDAR system, and the price is acceptable for many departments[1].

## Supplementary methods

### The details of waveform decomposition

Because the incident wave is a Gaussian beam, we can assume that the echo follows a Gaussian distribution and that the echo is isotropy; thus, the generalized gauss function is used as a kernel function for waveform decomposition[1]. Illumination of the waveform decomposition is as follows:

**Waveform denoising**. The median absolute deviation method is used to compute the system noise in the echo. For each echo signal $i$ from the filtered full-waveform data, the expression is:

$$\varphi_i = \eta \times \text{median}(|f_i(t)| - m), \ \text{m} = \text{median}\big(f_i(t)\big) \tag{1}$$

In this study, $\varphi_i$ is noise, $f_i(t)$ is the sampling intensity value in every echo, η is the consistency factor, and $\varphi_i$ is treated as an additive constant in the later waveform decomposition.

**Component detection.** After denoising, component detection is used to provide the initial state of the waveform decomposition for each echo signal $i$. Based on the nonlinear least squares fitting (NLSF), the initial estimates of the parameters are obtained. Then, the parameters vector $\vec{\theta}$ is computed by the iterative computations:

$$\vec{\theta} = argmin \sum_{i=1}^{n} \left(y_i - f\left(x_i|\vec{\theta}\right)\right)^2 \tag{2}$$

Here, $\vec{\theta}$=$\{a_j, \mu_j, \sigma_j, \alpha_j\}$, where $j$ is the component of every echo signal, $a_j$ is the amplitude of the component, $\mu_j$ is the location of the component, $\sigma_j$ is the pulse width of the component, $\alpha_j$ is the shape factor, $y_i$ is the intensity value of the echo, and $f\left(x_i|\vec{\theta}\right)$ is the NLSF function using the parameters. Thus, the initial parameters vector $\vec{\theta}$ can be calculated.

**Expectation Maximization Algorithm (EM).** For each echo signal $i$, the model (generalized gauss function) is initialized with $\vec{\theta}$=$\{a_j, \mu_j, \sigma_j, \alpha_j\}$, and the generalized gauss function can be expressed as:

$$y = \sum_{j=1}^{k} a_j exp\left(-\frac{|x-\mu_j|^{\alpha_j{}^2}}{2\sigma_j{}^2}\right), j = 1 \dots\dots k, \omega_j^{(0)}, \mu_j^{(0)}, \sigma_j^{(0)}, \alpha_j^{(0)} \tag{3}$$

In this study k is the number of components, $a_j$ is the amplitude of the component, $\omega_j^{(0)}$ is the initial weight of the component, $\mu_j^{(0)}$ is the initial location of the component, $\sigma_j^{(0)}$ is the initial pulse width of the component, $\alpha_j$ is the initial shape factor, x is the sampling location, and y is the intensity value of the echo.

Therefore, the expectation equation can be solved by:

$$L^{(0)} = \frac{1}{n}\sum_{i=1}^{n} log\left(\sum_{j=1}^{k} \omega_j^{(0)} \phi\left(y_i|\mu_j^{(0)}, \sigma_j^{(0)}, \alpha_j^{(0)}\right)\right) \tag{4}$$

Here, n is the amount of sampling in each echo.

Then, the expectation of the parameters vector $\vec{\theta}$ can be calculated as:

$$\gamma_{ij}^{(m)} = \frac{\omega_j^{(m)}\phi\left(y_i|\mu_j^{(m)},\sigma_j^{(m)},\alpha_j^{(m)}\right)}{\sum_{j=o}^{k}\omega_j^{(m)}\phi\left(y_i|\mu_j^{(m)},\sigma_j^{(m)},\alpha_j^{(m)}\right)}, i = 1 \ldots\ldots n, j = 1 \ldots\ldots k \quad (5)$$

$$n_j^{(m)} = \sum_{i=1}^{n}\gamma_{ij}^{(m)}, j = 1 \ldots\ldots k \quad (6)$$

and the next step can be calculated as:

$$\omega_j^{(m+1)} = \frac{n_j^{(m)}}{n}, j = 1 \ldots\ldots k \quad (7)$$

$$\mu_j^{(m+1)} = \frac{1}{n_j^{(m)}}\sum_{i=1}^{n}\gamma_{ij}^{(m)}y_i, j = 1 \ldots\ldots k \quad (8)$$

$$\sigma_j^{(m+1)} = \frac{1}{n_j^{(m)}}\sum_{i=1}^{n}\gamma_{ij}^{(m)}\left(y_i - \mu_j^{(m+1)}\right)\left(y_i - \mu_j^{(m+1)}\right)^T, j = 1 \ldots\ldots k \quad (9)$$

where m is the iteration. The iterative computation ends at the condition:

$$L^{(m)} = \frac{1}{n}\sum_{i=1}^{n} log\left(\sum_{j=1}^{k}\omega_j^{(m+1)}\phi\left(y_i|\mu_j^{(m+1)},\sigma_j^{(m+1)},\alpha_j^{(m+1)}\right)\right) \quad (10)$$

$$|L^{(m+1)} - L^{(m)}| < \varepsilon \quad (11)$$

Here, $\varepsilon$ is the threshold value, which can be an empirical number or the standard deviation of the residual error of the adjacent iterative computation.

According to the above calculation, the optimal solutions of the waveform features ($a_j$–echo amplitude, $\sigma_j$–echo width, $\mu_j$–echo order) are obtained for classification.

**The details of radiometric correction**

Because different echoes have different distances between the object and laser scanner, the radiometric correction is used to remove the distance effect on the echo energy[2]. The theory is derived primarily from the radar equation, and the laser energy equation can be expressed as an integral for the laser scanner:

$$P_r(t) = \frac{D^2}{4\pi\lambda^2}\int_0^H \frac{\eta_{sys}\eta_{atm}}{R^4}P_t\left(t - \frac{2R}{V_g}\right)\sigma(R)dR \quad (12)$$

where t is the time; D is the aperture diameter of the receiver optics; Pr is the received

power; $P_t$ is the emitted power; $\lambda$ is the wavelength; H is the scanner's height; R is the distance from the system to the target; $\eta_{atm}$ and $\eta_{sys}$ are, respectively, the atmospheric and system transmission factors; vg is the group velocity of the laser pulse; and $\sigma$(R)dR is the apparent effective differential cross-section. This equation describes the changes of the received power in a dynamic process. Then, we arrive at the following static formulation of the radar equation[2]:

$$P_r = \frac{P_t {D_r}^2}{4\pi R^4 {\beta_t}^2} \eta_{sys} \eta_{atm}\, \sigma \tag{13}$$

$$\sigma = \frac{4\pi}{\Omega} \rho A_s \tag{14}$$

$$A_s = \frac{\pi R^2 \beta_t^2}{4} \tag{15}$$

where $\beta_t$ is the beamwidth of the incident laser beam, $\Omega$ is the cone of the solid angle, $\sigma$ is the cross-section, $\rho$ is the reflectance of an object, and $A_s$ is the approximate footprint area of the beam at the scatterer.

From equations (13)-(15), $P_r$ is shown to follow the $1/R^2$ and $\rho$ laws. For an airborne laser scanner (ALS), R can be approximately expressed as:

$$\mathrm{R} = \mathrm{H} - \Delta\mathrm{R} \tag{16}$$

where H is the flying altitude of the ALS system and $\Delta$R is the distance between the object and the ground. When $\mathrm{H} \gg \Delta\mathrm{R}$, the effect of $1/R^2$ can be ignored. However, for the terrestrial laser scanner (TLS) in this study, the influence of $1/R^2$ must be considered for $P_r$.

Previous studies on the TLS radiometric correction and calibration clearly state that the range dependence of the TLS amplitude and intensity does not entirely follow the $1/R^2$ law of the radar equation, in particular, at near distances; the reasons for this

dependence may be detector effects (e.g., the brightness reducer, amplification, or gain control) or receiver optics (a defocusing and incomplete overlap of the beam and receiver's field of view). However, most manufacturers do not provide enough insight into developing a model-driven correction for these effects[2]. For the scanner (Riegl VZ-4000) used in this study, the $1/R^2$ correspondence is provided for distances >30 m. The incident angle $\vartheta$ affects the $P_r$ for the TLS[3], and the approximate relationship follows the cos $\vartheta$ law; therefore, the cos $\vartheta$ can be treated as 1 when the object is located at far range. In this study, the scanner is located 35 m from the nearest marine debris object; thus, we can follow the $1/R^2$ law and ignore the effect of the incident angle $\vartheta$ to make a radiometric correction based on the simple radiometric correction equation:

$$P_i' = \frac{R_i^2}{R_d^2} P_i \qquad (17)$$

In this study, $P_i$ is the energy of each echo i, $R_i$ is the range of each echo i, $R_d$ is range of each data point, and $P_i'$ is the normalized energy of $P_i$ (corrected to the datum point). Because we regard the echo as a general Gaussian wave, each echo's amplitude is multiplied by $R_i^2/R_d^2$ for the radiometric correction.

**The details of SVM**

We use LIBSVM which is available at http://www.csie.ntu.edu.tw/~cjlin/libsvm in this study. LIBSVM can optimize the penalty factor C and the inner parameter gamma in kernel function for classification, and it is very suitable for this study.

**The details of threshold value**

The threshold value is set to reduce the influence of misclassified points coming from the SVM classifier. Most of misclassified points are located at the edge of an object

because the laser footprint does not entirely touch the edge of the object. For an incident laser at the edge of object, part of its energy can pass onward to the next object so that the echo contains a certain amount of undesired signal, which may lead to misclassification in the SVM classifier. Those misclassified points can be mistaken for the ground or other types of marine debris. In the individual separation step, the misclassified points would be separated into fictitious single objects, which in reality do not exist; therefore, we can ignore these fictitious single objects to greatly improve the accuracy of the marine debris quantity statistics.

The threshold value is an empirical number, which is primarily determined via spatial resolution and range variation changes. For example, the threshold value changes from 100 to 15 with a range from 70 m to 150 m at Nanhui beach. We tend to identify as many small objects as possible while reducing as many misclassified points as possible.

**Supplementary tables**

Table S1. Confusion matrix of SVM in control experiment on Nanhui beach (%).

| | Plastic | Paper | Cloth | Metal | Ground |
|---|---|---|---|---|---|
| Plastic | 91.41 | 6.55 | 2.28 | 3.43 | 6.65 |
| Paper | 2.11 | 80.82 | 4.77 | 4.39 | 5.35 |
| Cloth | 4.56 | 4.18 | 79.72 | 5.11 | 5.88 |
| Metal | 1.03 | 5.31 | 7.42 | 77.84 | 10.23 |
| Ground | 0.89 | 3.14 | 5.81 | 9.23 | 71.89 |

**Supplementary Figures**

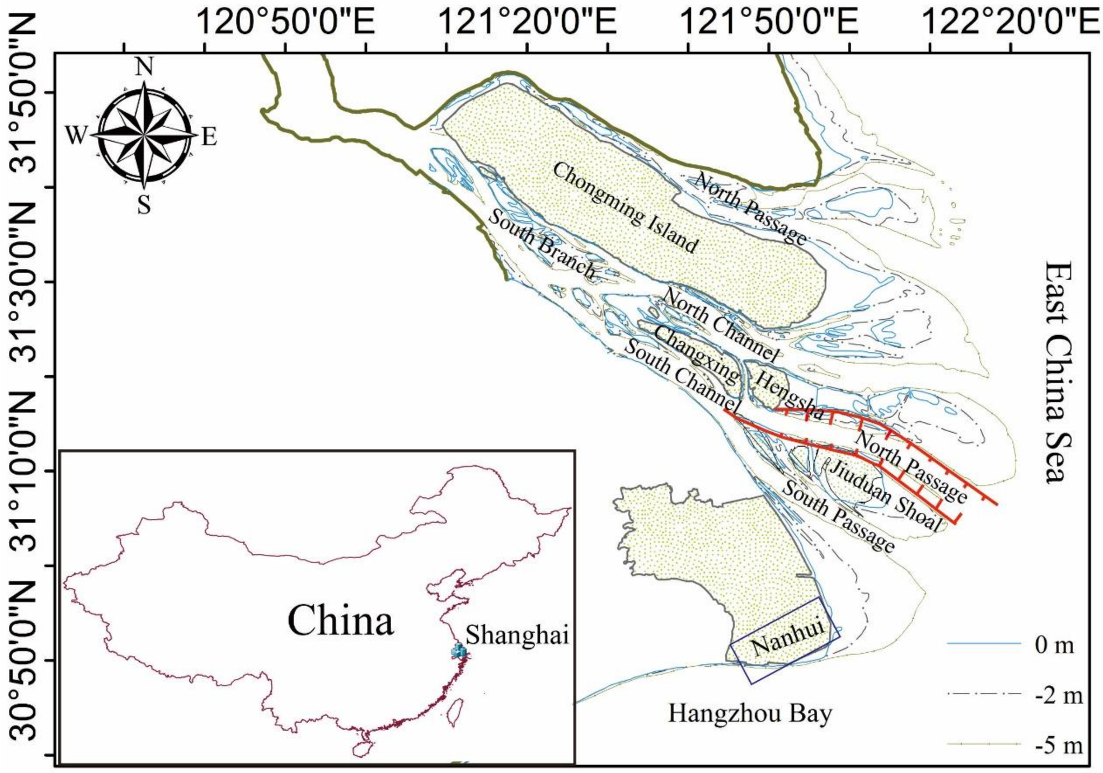


Fig. S1. The location of the research in Nanhui Beach. The figure was created with ArcGIS 10.1 (http://www.esri.com/).

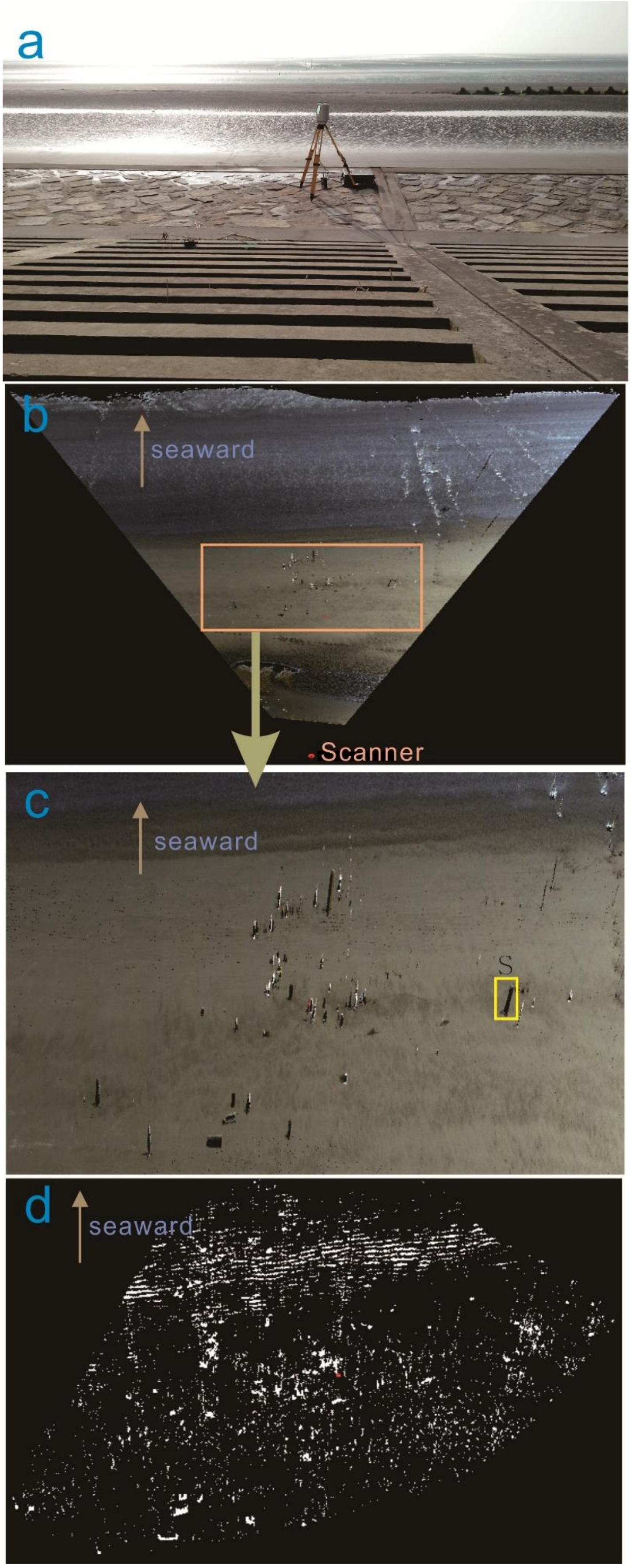


Fig. S2. Data acquisition and elevation filtering. (a) Scanner deployment at Nanhui

beach. (b) 2D view of point-cloud data. (c) Extracted part from the green box in (b). The black part (similar to the yellow box S) signifies no data in (c). (d) Residual points after elevation filtering. Benefiting from the camera and laser scanner, both (b) and (c) are shown with color. The color is only the visual effect, and it doesn't participate in extracting marine debris. The photo was taken by Ge, Z.P. at Shanghai on 1 January, 2015. The figure was created in RiSCAN PRO 1.7.8 (http://www.riegl.com/) and CorelDRAW Graphics Suite X5 (http://www.coreldraw.com/cn/).

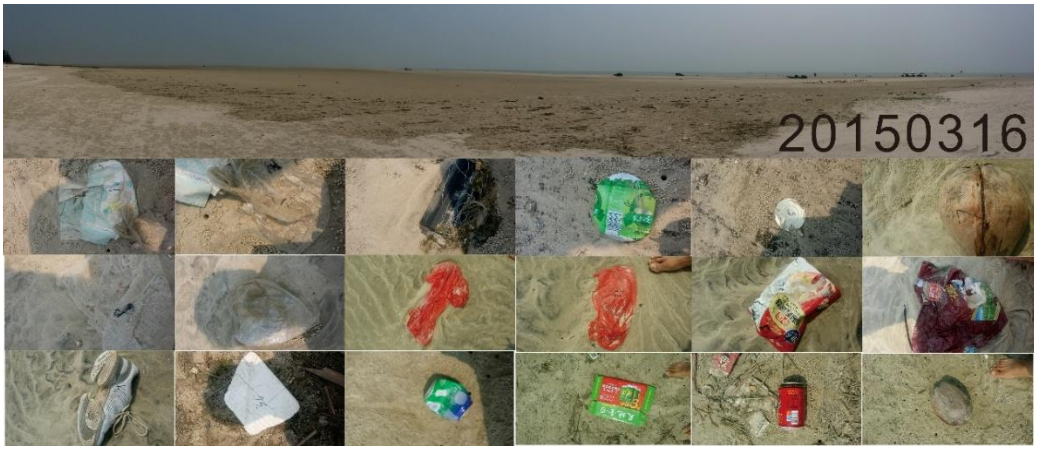


Fig. S3. Photos of some of the debris at Beihai beach on 2015.3.16. The photos were taken by Ge, Z.P. The figure was created in CorelDRAW Graphics Suite X5 (http://www.coreldraw.com/cn/).

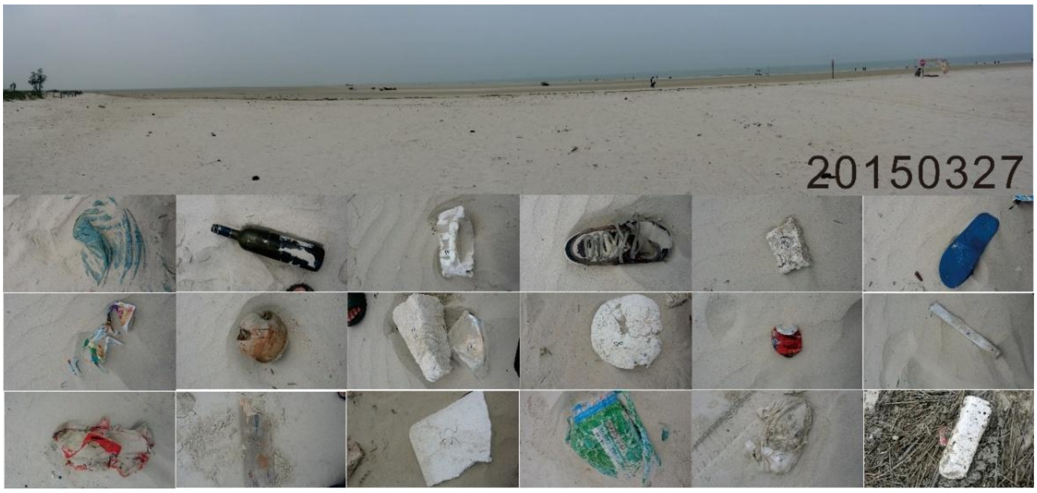


Fig. S4. Photos of some debris on Beihai beach at 2015.3.27. The photos were taken by Ge, Z.P. The figure was created in CorelDRAW Graphics Suite X5 (http://www.coreldraw.com/cn/).

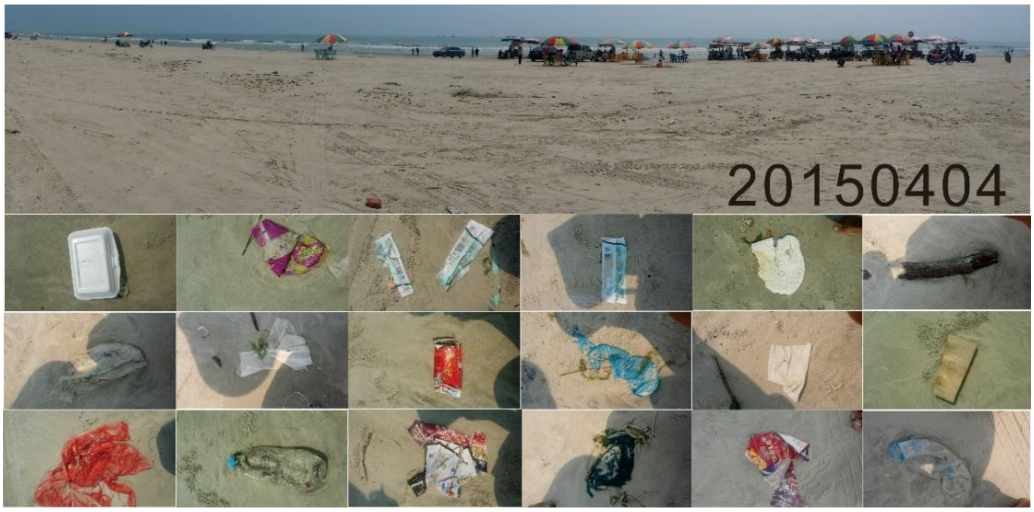


Fig. S5. Photos of some debris on Beihai beach at 2015.4.4. The photos were taken by Ge, Z.P. The figure was created in CorelDRAW Graphics Suite X5 (http://www.coreldraw.com/cn/).

## Supplementary References


[1] Höfle, B. Radiometric correction of terrestrial LIDAR point cloud data for individual maize plant detection. *IEEE Geosci. Remote Sens. Lett.* **11 (1)**, 94–98 (2014).

[2] Wagner, W. Radiometric calibration of small-footprint full-waveform airborne laser scanner measurements: Basic physical concepts. ISPRS J. Photogramm. Remote Sens. **65**, 505-513

(2010).

[3] Kaasalainen, S., et al. Analysis of Incidence Angle and Distance Effects on Terrestrial Laser Scanner Intensity: Search for Correction Methods. *Remote Sens.* **3**, 2207-2221 (2011).
